# Owner reports of attention, activity, and impulsivity in dogs: a replication study

**DOI:** 10.1186/1744-9081-6-1

**Published:** 2010-01-04

**Authors:** Lisa Lit, Julie B Schweitzer, Ana-Maria Iosif, Anita M Oberbauer

**Affiliations:** 1 MIND Institute and Department of Neurology, University of California at Davis, Sacramento, CA, USA; 2 Department of Psychiatry and Behavioral Science, University of California at Davis, Sacramento, CA, USA; 3 Department of Public Health Sciences, Division of Biostatistics, University of California at Davis, Davis, CA, USA; 4 Department of Animal Science, University of California at Davis, Davis, CA, USA

## Abstract

**Background:**

When developing behaviour measurement tools that use third party assessments, such as parent report, it is important to demonstrate reliability of resulting scales through replication using novel cohorts. The domestic dog has been suggested as a model to investigate normal variation in attention, hyperactivity, and impulsive behaviours impaired in Attention Deficit Hyperactive Disorder (ADHD). The human ADHD Rating Scale, modified for dogs and using owner-directed surveys, was applied in a European sample. We asked whether findings would be replicated utilizing an Internet survey in a novel sample, where unassisted survey completion, participant attitudes and breeds might affect previous findings.

**Methods:**

Using a slightly modified version of the prior survey, we collected responses (*n *= 1030, 118 breeds representing 7 breed groups) primarily in the United States and Canada. This study was conducted using an Internet survey mechanism.

**Results:**

Reliability analyses confirmed two scales previously identified for dogs (inattention [IA], hyperactivity-impulsivity [HA-IM]). Models including age, training status, and breed group accounted for very little variance in subscales, with no effect of gender.

**Conclusions:**

The factor invariance demonstrated in these findings confirms that owner report, using this modified human questionnaire, provides dog scores according to "inattention" and "hyperactivity-impulsivity" axes. Further characterization of naturally occurring variability of attention, activity, and impulsivity in domestic dogs may provide insight into genetic backgrounds underlying behaviours impaired in attention and associated disorders.

## Background

Attention deficit hyperactivity disorder (ADHD), a neurodevelopmental disorder characterized by inattention, hyperactivity, and impulsivity, has a prevalence rate of 3-7% in children [1] and over 4% in adults [2]. The disorder, diagnosed primarily through parent and teacher report, is associated with impaired social relationships and academic functioning, as well as increased substance abuse, risk-taking behaviour, and underemployment [3]. Though twin concordance studies support high heritability, global susceptibility genes have not been identified, and non-overlapping genetic studies implicate multiple genes and encompass multiple chromosomal regions [[4], e.g. [5], reviewed in [6]].

An appropriate animal model for ADHD must demonstrate face (similar behavioural characteristics), construct (theoretical rationale), and predictive (correctly predict behavioural and biological characteristics) validity [7]. More specific criteria described for face validity of animal models for ADHD include impulsivity that develops over time, inattention with temporally widely-spaced stimuli, and hyperactivity in a non-novel environment that develops over time; ADHD-specific criteria for construct validity include altered reinforcement of novel behaviour and deficient extinction of previously reinforced behaviour [7].

Although non-human primates exposed to the dopamine neurotoxin 1-methyl-4-phenyl-1,2,3,6-tetrahydropyridine (MPTP) have been suggested as appropriate ADHD models [8-10], animal models for ADHD have historically been rat or mouse models [11]. Rodent models offer advantages of genetic homogeneity, relatively low cost, ease of generating and maintaining large populations for research, and control of environmental variables [11]. An additional advantage is a simpler nervous system offering similar basic behavioural mechanisms.

The spontaneously hypertensive rat (SHR), a rodent model demonstrating face, construct and predictive validity for ADHD, displays inattentive, hyperactive and impulsive behaviours [12]. These ADHD-like behaviours are genetic rather than environmental, as demonstrated by lack of effects of cross-fostering [13] or social isolation [14]. The SHR also demonstrates some sex differences similar to those observed in ADHD in humans [15]. There have been reports questioning the validity of the SHR, particularly when compared with the Wistar-Kyoto (WKY) strain typically used as a control [16-18]. However, further investigations have demonstrated that distinct strains are more representative of different subtypes of ADHD, and that insufficient consideration of substrain variability together with paradigm application might underlie concerns about SHR validity as an ADHD model [[19], reviewed in [20]].

Additional rodent models for ADHD have been created by genetic engineering to generate an ADHD-like phenotype, such as the SNAP-25 deficient mouse mutant coloboma [[21-23], reviewed in [24]] and the dopamine transporter knockout mouse (DAT-KO) [e.g. [25,26]]. Other models have been developed through experimental exposure to central nervous system insult during prenatal or postnatal development, for example the anoxic mouse [27,28], rearing in social isolation [29], and exposure to environmental toxins such as polychlorinated biphenyls and lead [30,31]. Unlike the SHR, these models do not recapitulate all symptoms of ADHD and thus fail to meet criteria for validity as previously described [7]. Despite this, some overlap has been found across models; for example, overlaps in neurotransmitter-related and epigenetic gene expression levels were recently identified in both SHR and PCB-exposed Sprague-Dawley rats [32]. Therefore these additional models might offer complementary information to help tease apart the behavioural and biological heterogeneity found in ADHD.

Recently, the domestic dog has been proposed as an innovative genetic and behavioural model for complex behaviours in humans [33-37], including ADHD [38-40]. The dog model has the potential to provide an important additional animal model in the quest to understand ADHD, supplementing rather than replacing existing animal models. Genetically, the dog is less diverged from the human than the mouse [41], with dogs and humans having approximately the same number of genes, most of which descended from the same ancestral genes [34]. Behaviourally, dogs demonstrate heritable social cognitive behaviours in response to both conspecific and human cues [reviewed in [42]], often exceeding capabilities of non-human primates [43,44]. The predictive validity of a dog model is further enhanced by observed effects of human psychopharmacological agents used in dogs for behaviours phenotypically similar to those seen in human psychopathologies, such as obsessive-compulsive disorder and generalized anxiety disorder [45]. This suggests that similar underlying biological mechanisms contribute to homologous behaviours across dogs and humans.

Modified human measurement scales have been applied successfully to evaluate dog behaviours [e.g. [46]], including behaviours relevant to ADHD. The Du Paul ADHD-Rating Scale (ADHD-RS) [47] is based on the DSM-IV criteria for ADHD and is composed of two subscales, inattention and hyperactivity/impulsivity. This scale, modified for dogs and using owner-directed surveys, was recently validated using Eastern European dog owners [40]. Although pathological levels of inattention, hyperactivity or impulsivity have not been established in dogs, these scales may be useful for examining genetic contributions to variability of the represented behaviours in dogs. For example, subscales have subsequently been used to consider the association between polymorphisms in genes that have been implicated in human ADHD and variation in subscale scores in dogs [38,39].

However, because parent report measures in humans can be subject to bias based on many factors, such as cultural background [48,49], it is possible that survey findings of Vas et al. [40] might be influenced by regional beliefs about dog behaviour, training practices, and preferred dog breeds. In addition, the previous surveys were completed with researcher assistance if necessary [40]. Thus, we questioned whether comparable findings could be obtained using a novel population of dog owners, in the absence of researcher assistance to complete the survey.

Our study does not address the question of whether dogs with extreme scores display evidence of a medical or behavioural problem [50,51], or whether the dog is an appropriate animal model for pathologies reflected in ADHD based on this questionnaire [50,52]. The primary purpose of this study is to expand the findings in Vas et al. [40] by utilizing an unassisted Internet survey targeting dog owners, to investigate whether different beliefs about dog behaviour, breeds represented, and training practices might result in markedly different response profiles than those in the original Eastern European sample. Similar findings would underscore the reliability of owner report for these behaviours in their dogs, as well as validate Internet survey data collection for observed attention, activity, and impulsivity related behaviours of the dog.

## Methods

### Dog-ADHD rating scale

A modification of the dog-ADHD rating scale owner version used by Vas et al. [40] was used (Additional file 1). The question "It is excessive, difficult to control, if it lunges it is hard to hold back." was removed as pilot studies suggested that this question was highly dependent on training and equipment. Specifically, the increased utilization of training equipment that typically offers immediate control over the behaviours described in this question (i.e., dog halters, no-pull harnesses) might substantially skew responses. Other questions were modified slightly to adjust for language differences. A 5-point Likert scale was used instead of a 4-point Likert scale (response choices were "Never/Rarely," "Occasionally," "Often," "Very Frequently," and "Always," scored as 0, 1, 2, 3, and 4 respectively). However, overall the revised rating scale in this report remained reflective of the ADHD Rating Scale, Parent version [47], with questions limited to those appropriate for dog behaviours. As in Vas et al. [40], two *a priori *subscales were initially defined: an inattention subscale (IA, questions 1, 2, 3, 7, 9, 11) and a hyperactivity-impulsivity subscale (HA-IM, questions 4, 5, 6, 8, 10, 12) (Additional file 1). The Institutional Review Board at the University of California at Davis approved the survey.

### Additional information

Respondents were asked to provide geographical region of residence, dog's breed, gender, spay/neuter status, birth date, medications, and health problems. Training and certification status of dogs were also collected, including whether dogs had basic pet dog training, certification by the American Kennel Club (AKC) in canine manners (Canine Good Citizen, CGC), and training/certification in competitive obedience, schutzhund, agility, therapy dog, search and rescue, scent detection, or other venues.

### Survey completion

The survey was completed via an Internet survey mechanism [53] (Additional file 1). A link to the survey was initially distributed via email to dog owners and groups provided by the authors, as well as posted on several dog-related training and pet groups. To avoid an inherent selection bias in those exposed to the survey, efforts were made to target pet, sport, and professional dog owners and handlers. Pilot studies indicated that survey completion took between 5 and 10 minutes. Survey data were collected between September 2008 and January 2009.

### Participants

Sample demographics are summarized in Table 1. Initial survey responses included 1184 dogs. Dogs were excluded (*n *= 46) if age data or training background data were missing, or if dog age was greater than 203 months. The age cutoff of 203 months includes dogs less than 17 years of age at the time the owner completed the survey. Specifically, seven dogs were omitted using this criterion. Of these, three were also missing data, and were excluded on that basis. Ages of remaining dogs ranged from 19 to 43, appeared as an extreme tail in the distribution of dog ages, potentially represented erroneous responses, and were eliminated. Samples with missing responses on the ADHD Rating Scale survey were also excluded (*n *= 108), yielding a final analysis sample of 1030 dogs. Responses were received predominantly from the United States (*n *= 980) and from Canada (*n *= 41). Responses were also received from Australia, England, Germany, New Zealand, Portugal, and Scotland (*n *= 9 total) (Table 1). Because these nine responses represented countries not included in Vas et al. [40], they were retained for analysis. Mean age in months (± SEM) of all dogs included (*n *= 1030) was 75.8 ± 1.4 months.

**Table 1:** Sample demographics, n = 1030 dogs.

Age groups	*n*	*(mean ± SE)*
Mean age, all dogs	1030	(75.8 ± 1.4)
Juvenile (4.5-23 months)	129	(15.6 ± 0.5)
Young Adult (24-59 months)	301	(42.7 ± 0.6)
Adult (60-95 months)	260	(77.1 ± 0.6)
Senior (96-23 months)	340	(126.9 ± 1.3)
**Geographic distribution of responses**		**(%)**
(49) US states represented (incl. District of Columbia)	980	(95.15)
Canada	41	(3.98)
(7) Countries other than United States	9	(0.87)
**Gender distributions**		
Males	531	(51.6)
Neutered males	392	(38.1)
Intact males	139	(13.5)
Females	495	(48.1)
Spayed females	408	(39.6)
Intact females	87	(8.4)
**Breed information**		
Number of AKC breeds represented	118	
**Distribution of dogs by AKC group**		
Herding	260	(25.2)
Hound	51	(5.0)
Non-sporting	85	(8.3)
Sporting	205	(19.9)
Terrier	56	(5.4)
Toy	37	(3.6)
Working	70	(6.8)
Not AKC	51	(5.0)
Mixed breed	205	(19.9)
**Training status**		
Untrained	288	(28.0)
Beginner	289	(28.1)
Advanced	453	(44.0)

^1^Note: age was used as a continuous covariate in analyses. As such, Age Groups are provided for informational purposes only.

Based on owner report of dog breeds, 118 AKC-recognized breeds were represented (Additional file 2). These were further stratified according to AKC group (Herding, Hound, Non-sporting, Sporting, Terrier, Toy, and Working) (Table 1). Additionally, 51 dogs were non-AKC recognized purebreds, and 205 dogs were mixed-breed.

To assign Training Status, dogs with only pet dog training and/or training for a CGC title were considered "Untrained", dogs with either a CGC title or training in a working discipline were defined as "Beginner", and dogs with at least one title in a working discipline were considered to have "Advanced" training (Table 1).

### Statistical analyses

Analyses were based on those performed by Vas et al[40]. IA and HA-IM subscale scores were calculated for each dog by adding scores for questions within each subscale. Cronbach's alpha was used to examine reliabilities of *a priori *subscale assumptions [54,55]. Independence of subscales was evaluated with Pearson correlations. Factor analysis using varimax rotation was used on all dog-ADHD survey questions to identify factors and loadings of individual items, with results compared to *a priori *subscale definitions.

Effects of Age, Neuter Status, Gender, Breed Group, and Training Status on subscale scores were evaluated with analysis of variance (ANOVA) or covariance (ANCOVA) models, as appropriate. Bonferroni corrections were used in pairwise comparisons to adjust for multiple comparisons.

Significance of predictors was assessed using two-sided tests with *α *= 0.05. SPSS 17.0 was used for all analyses [56].

## Results

### Reliability of a priori subscales

The *a priori *IA subscale with six questions (questions 1, 2, 3, 7, 9, 11, Additional file 1) had good internal reliability, *α *= 0.88. All items appeared worthy of retention, as Cronbach's alpha would not measurably improve with exclusion of any item (Table 2, "Cronbach's alpha if item deleted" column). All items correlated with the total subscale to a good degree (lower *r *= 0.42) (Table 2, "Corrected item-Total Correlation" column).

**Table 2:** For each a priori subscale, Cronbach's alpha and subscale component reliabilities.

	Mean	Standard Deviation	Corrected item - Total Correlation	Cronbach's alpha if item deleted
*Inattention subscale *(alpha = 0.85)				
1. Other things attract attention	1.81	0.81	0.73	0.80
2. Loses interest easily	1.79	0.80	0.67	0.82
3. Difficulty concentrating	1.48	0.70	0.71	0.81
7. Doesn't pay attention to someone speaking to him/her	1.40	0.71	0.42	0.86
9. Difficulty performing practiced tasks	1.41	0.68	0.58	0.83
11. Easily distracted	2.02	0.88	0.70	0.81
				
*Hyperactivity-Impulsivity subscale *(alpha = 0.67)				
4. Difficulty maintaining stay	1.91	0.89	0.36	0.63
5. Barks endlessly	1.36	0.75	0.30	0.66
6. Fidgets or in constant motion	1.54	0.95	0.52	0.58
8. Likes active play/running around	3.59	1.23	0.26	0.69
10. Reacts hastily/anticipates	2.15	1.00	0.48	0.59
12. Cannot wait	1.55	0.81	0.54	0.58

The *a priori *hyperactivity-impulsivity subscale (HA-IM) with six questions (questions 4, 5, 6, 8, 10, 12, Additional file 1) had lower internal reliability, *α *= 0.67. As with the IA subscale, there would be very little improvement with exclusion of any item for the HA-IM subscale (Table 2, "Cronbach's alpha if item deleted" column). There was lower correlation of individual items with the total subscale (lower *r *= 0.26). Overall, the reliability analysis of the HA-IM scale (*α *< 0.7) suggested the possible existence of additional subscales within that scale [57].

### Factor analysis

To explore dimensionality of scale items and whether additional constructs could account for patterns of item scores, factor analysis with varimax rotation was performed using all 12 questions in the dog ADHD rating scale as input (Additional file 1). Using the criterion of eigenvalues greater than 1.0 [58,59], three factors, identified as Inattention (IA, M = 0.19, SE = 0.004, range = 0.00-0.62), Hyperactivity-Impulsivity 1 (HY1, M = 0.18, SE = 0.004, range = 0.00-0.70), and Hyperactivity-Impulsivity 2 (HY2, M = 0.36, SE = 0.004, range = 0.14-0.70), were extracted accounting for 59.2% of the total variance (Table 3).

**Table 3:** Principal component analysis (varimax rotation) factor loadings.

	Subscale		
	Inattention	Hyperactivity-Impulsivity 1	Hyperactivity-Impulsivity 2
1. Other things attract attention	**0.819**	0.098	0.198
2. Loses interest easily	**0.831**	0.036	-0.009
3. Difficulty concentrating	**0.784**	0.248	-0.025
7. Doesn't pay attention to someone speaking to him/her	**0.451**	0.357	-0.111
9. Difficulty performing practiced tasks	**0.596**	0.382	0.099
11. Easily distracted	**0.774**	0.147	0.300
4. Difficulty maintaining stay	0.387	**0.484**	0.147
5. Barks endlessly	0.072	**0.801**	-0.030
6. Fidgets or in constant motion	0.074	**0.543**	**0.530**
12. Cannot wait	0.378	**0.554**	0.344
8. Likes active play/running around	-0.046	-0.093	**0.820**
10. Reacts hastily/anticipates	0.213	0.207	**0.689**

The first factor, IA (eigenvalue = 4.57), included the six questions comprising the *a priori *inattention subscale and accounted for 38.1% of total variance. The second factor, HY1 (eigenvalue = 1.51), accounted for 12.6% of total variance and included four of the six questions represented in the HA-IM scale. The remaining two questions from the *a priori *HA-IM scale are found in factor three, HY2 (eigenvalue = 1.02), accounting for 8.5% of total variance. These two questions (question 8 and question 10, Additional file 1) had high loadings on this factor (0.82 and 0.69 respectively). In addition, question 6 loaded across both the second (0.54) and third (0.53) factors, and subsequently was included in the calculation of both subscales. Thus, the *a priori *HA-IM scale was split, so that questions 4, 5, 6, and 12 comprised the Hyperactivity-Impulsivity scale 1 (HY1), and questions 6, 8, and 10 were used to create a Hyperactivity-Impulsivity scale 2 (HY2) (Additional file 3).

We then compared the *a priori *subscale scores for inattention and hyperactivity/impulsivity (described above) with factor scores generated through factor analysis. As expected, subscale scores and factor scores were significantly and highly correlated (IA-Factor 1: *r *= 0.95, HY1-Factor 2: *r *= 0.83, HY2-Factor 3: *r *= 0.94, all *p *values < 0.0001). As found in Vas et al. [40], the strong relationships between *a priori *subscale scores and factor scores suggest that Factor 1 is associated with attention and that Factors 2 and 3 are related to activity and impulsive behaviours.

Finally, because both IA and HY1 subscales demonstrated slight positive skew and kurtosis, a logarithmic transformation was applied. To allow comparison across subscales, the logarithmic transformation was also applied to the HY2 subscale. Means and standard deviations for transformed subscales were: IA 0.20 ± 0.14, HY1 0.18 ± 0.14, and HY2 0.36 ± 0.14. As expected from loading patterns, subscales were not independent. IA was correlated with both HY1 (*r *= 0.51, *p *< 0.001) and HY2 (*r *= 0.24, *p *< 0.001), and HY1 and HY2 were correlated (*r *= 0.51, *p *< 0.001).

### Age

Age in months was calculated based on birth dates provided for dogs. There was an association between Age in months and Training Status (*r *= 0.14, *p *< 0.0001), with older dogs more likely to have more training.

Additionally, Age in months was a significant predictor of IA, HY1, and HY2 (all *p *values < 0.001); older dogs had lower subscale scores. Although Age accounted for very little variance in each subscale (*R*^2 ^IA: 0.02, HY1: 0.02, HY2: 0.08), it was included as a covariate in subsequent analyses.

### Univariate analyses

Univariate ANCOVAs controlling for Age were performed to assess the effect of each qualitative demographic independent variable (Gender, Neuter Status, Breed Group, and Training Status) on subscales.

Effects of Gender and Neuter Status were evaluated using a 2 × 2 (Gender [Male, Female] × Neuter Status [Neutered, Intact]) factorial ANCOVA. There was a main effect of Gender on HY1 (*p *= 0.02, *η*^2 ^= 0.01); females had higher mean HY1 scores than males (Table 4). There was a very small but significant main effect of Neuter Status on IA (*p *= 0.03, *η*^2 ^= 0.005), with neutered dogs (M = 0.20, SE = 0.01) having higher IA scores than Intact dogs (Table 4). There was no interaction between Gender and Neuter Status.

**Table 4:** Raw means and standard errors (SE) for Breed Group, Training Status, Gender, and Neuter Status.

	Inattention	Hyperactivity-Impulsivity 1	Hyperactivity-Impulsivity 2
	
	Mean (SE)	Mean (SE)	Mean (SE)
**Breed Group**			
Herding	1.51 (0.03)	1.57 (0.04)	2.58 (0.05)
Hound	1.98 (0.10)	1.88 (0.11)	2.39 (0.11)
Non-sporting	1.70 (0.06)	1.59 (0.07)	2.28 (0.08)
Sporting	1.58 (0.04)	1.54 (0.04)	2.36 (0.06)
Terrier	1.76 (0.07)	1.57 (0.08)	2.45 (0.10)
Toy	1.84 (0.11)	1.71 (0.12)	2.22 (0.15)
Working	1.58 (0.14)	1.46 (0.07)	2.32 (0.08)
Not AKC	1.74 (0.08)	1.76 (0.09)	2.52 (0.10)
Mixed	1.72 (0.04)	1.58 (0.04)	2.40 (0.05)
**Training Status**			
Untrained	1.82 (0.04)	1.80 (0.04)	2.37 (0.05
Beginner	1.69 (0.03)	1.60 (0.03)	2.54 (0.05)
Advanced	1.52 (0.02)	1.45 (0.03)	2.38 (0.04)
**Gender**			
Female	1.65 (0.03)	1.62 (0.03)	2.45 (0.04)
Male	1.65 (0.03)	1.56 (0.03)	2.41 (0.03)
**Neuter Status**			
Intact	1.61 (0.04)	1.55 (0.04)	2.48 (0.05)
Spayed/Neutered	1.66 (0.02)	1.60 (0.02)	2.41 (0.03)

There were differences in both IA and HY1 subscales according to Training Status (IA: *p *< 0.001, *η*_*p*_^2 ^= 0.049, HY1: *p *< 0.001, *η*_*p*_^2 ^= 0.064) (Table 4). Bonferroni corrected pairwise comparisons indicated that for both IA and HY1 subscales, Untrained dogs had higher mean scores than both Beginner and Advanced dogs (Table 4), and Beginner dogs had higher mean scores than Advanced dogs (Table 4).

There were significant differences in mean IA, HY1, and HY2 subscale scores (Table 4) according to Breed Group (IA: *p *< 0.001, *η*_*p*_^2 ^= 0.055, HY1: *p *= 0.01, *η*_*p*_^2 ^= 0.023, HY2: *p *= 0.01, *η*_*p*_^2 ^= 0.022). For IA, dogs in the Hound, Terrier, Toy, and Mixed Breed groups each had higher mean scores than dogs in the Herding group (Table 4). Dogs in the Hound group also had higher mean IA scores than dogs in the Sporting group (Table 4). For HY1 scores, dogs in the Hound group had higher mean HY1 scores than dogs in the Herding, Sporting, and Working groups (Table 4). However, for HY2, dogs in the Herding group had higher mean HY2 scores than dogs in the Non-sporting and Toy groups (Table 4).

### Adjusted analyses

To evaluate contributions of Gender, Breed Group and Training Status predicting each of the three subscales, adjusted models were fitted for each subscale controlling for Age and using dog Gender, Training Status, and Breed Group as independent variables. Interactions between the predictors were also considered. Because the effect size of Neuter Status on IA was extremely small (*η*^2 ^= 0.005), and inclusion would have reduced the power of the study, Neuter Status was not included in these analyses. Only terms that added significantly to the model were retained for each subscale. For the IA and HY1 subscales, there were main effects of Training Status (all *p *values < 0.0001) and Breed Group (IA: *p *< 0.0001; HY1: *p *= 0.022). For the HY2 subscale, there was only a main effect of Breed Group and the univariate model was retained. For all subscales, there was no effect of Gender when controlling for effects of Age.

Bonferroni-corrected pairwise comparisons for the IA and HY1 subscales using the model "Subscale score = (Age) × Training Status × Breed Group" showed that dogs in the Hound (M = 0.29, SE = 0.02) group had higher mean IA scores than dogs in the Herding (M = 0.18, SE = 0.01) and Sporting (M = 0.19, SE = 0.01) groups, and that Untrained (M = 0.25, SE = 0.01) dogs had higher IA scores than Advanced (M = 0.19, SE = 0.01) dogs. This was different for HY1 scores; dogs in the Hound (M = 0.22, SE = 0.01) group had higher mean HY1 scores than dogs in the Working (M = 0.15, SE = 0.02) group, and Untrained (M = 0.24, SE = 0.01) dogs had higher mean HY1 scores than both Beginning (M = 0.17, SE = 0.01) and Advanced (M = 0.14, SE = 0.01) dogs.

For all models, effect sizes remained in the very small range (all effect sizes less than 0.08, with adjusted *R*^2 ^for all models below 0.10). That is, Age, Training Status and Breed Group together accounted for a small amount of variance in IA, HY1, and HY2 subscales.

## Discussion

In this study, we used a version of the ADHD Rating Scale [47] modified for dog owners to complete about their dogs' behaviours [40]. We collected Internet survey responses from a predominantly North American sample of dog owners. Our findings confirmed and expanded those of Vas et al. [40]. In our survey, owner reports of attention, activity, and impulsivity in dogs were unaffected by Gender when controlling for Age in adjusted models. In all models, the variables Age, Breed Group, and Training Status accounted for very little variance.

Our findings also confirmed that data collected with an Internet survey generated consistent findings when compared to researcher-assisted data collection, supporting web-based surveys as a means to generate valid data [60]. The attention subscale was particularly robust both in our data set and in Vas et al. [40]. Although two *a priori *subscales (IA and HA-IM) were previously identified [40], we identified a third subscale using factor analysis, suggesting that the HA-IM subscale might consist of two separable subscales. Indeed, HY2 was unaffected by Training Status, while both IA and HY1 showed effects of Training Status, with advanced training generating lower IA and HY1, but not lower HY2 subscale scores. Therefore this additional subscale might offer a valuable alternative endophenotype for subsequent investigations into genetic networks underlying attention, hyperactivity and impulsivity.

Consistent with Vas et al. [40], our complete model did not indicate any effect of physical size on IA, as the Toy group was not significantly different than other breed groups for any IA. However, we did find that the Herding group had significantly higher mean HY2 subscale scores than the Toy group. This differed from previous findings, and may demonstrate a difference identified by the presence of the additional subscale. The lack of effect of Gender on any subscale in dogs when effects of Age were controlled was consistent with previous findings [40,61].

We did find that some differences identified in univariate models were no longer present in adjusted models. This suggests that although the interactions between Breed Group and Training Status were not significant, they each accounted for sufficient variation in the other that the models including both variables were able to refine results. Even then, effect sizes remained extremely small. Differences in raw means (Table 4) were small, suggesting that statistically significant differences were due primarily to our large sample, and there are factors other than breed group, group, training, gender and age that account for variation in attention, activity and impulsivity. These factors may be environmental factors not captured by training history, such as living environment of the dog (outside vs. inside), amount of time dogs spend with their owners and activities other than training during that time or number of dogs in each household. It is also possible that variance can be explained by genetic background of dogs, but that comparing breed groups does not provide sufficient resolution to identify genetic variation that might be better identified through individual breed comparisons or across individuals within a single breed [38,39]. Moreover, assignment of training status according to certification or title received might result in some highly trained yet uncertified dogs included in the "untrained" category.

## Limitations

One limitation of this study is the need to confirm whether additional variance can be explained by environmental or genetic factors. In addition, usefulness of the third scale identified within this study is unclear, and future experiments will be required to clarify this. Importantly, behavioural and physiological measures that correlate with survey reports are needed to establish validity of owner perceptions when evaluating behaviours in their dogs. This study also illustrated that although large samples may be obtained through use of Internet survey data collection methods, incomplete data may substantially impact final sample sizes.

One objection to utilizing dogs as a model for social cognition and cognitive disorders has been the suspicion that anthropomorphism underlies interpretation of findings [42,62]. It is possible that this questionnaire is simply measuring human bias in reporting and perception; that is, the same questions will yield the same structure of responses, regardless of population. This is a problem reflected in any psychological survey measure used in humans, and underscores the need to develop understanding of biological bases of both normal and pathological behaviours, potentially through the use of novel animal models.

Alternatively, it might be argued that the modifications made to the questionnaire in this study compared with Vas et al. [40] affect whether we have really tested the generalizability of the previous work in a new population or simply examined a variant of it. However, the congruence of our findings in a predominantly North American population of dog owners with those in an eastern European population [40], particularly given the questionnaire modifications, confirm that response structure appears consistent across populations, irrespective of data collection mechanism, breeds, and training beliefs and practices.

## Conclusions

These findings confirm reliability of this questionnaire as a tool for capturing owner observations of variability in attention, activity levels, and impulsivity in dog populations, and demonstrate that owner report provides dog scores according to "inattention" and "hyperactivity-impulsivity" axes. Validation of the dog as an animal model for ADHD will require in-depth characterization of behavioural and biological measures, both within and across breeds. It will be necessary to confirm whether a group of dogs can be identified displaying impulsivity that develops over time, inattention with temporally widely-spaced stimuli, hyperactivity in a non-novel environment that develops over time, altered reinforcement of novel behaviour and deficient extinction of previously reinforced behaviour. Further validation might identify breeds or lines with breeds that are more representative of distinct subtypes of ADHD, as shown by SHR substrains [19,63]. It is also important to identify whether dogs with extreme scores on any subscale demonstrate behavioural pathology as well as how well owner report (like parent report) accurately reflects behaviour; however, those questions are beyond the scope of this study. Findings of our study suggest that information provided by this behaviour scale can reliably provide an important basis for considering the genetic underpinnings of and environmental contributors to these behaviours in a dog model. Overall, this consistency supports use of a modified human measurement tool to evaluate attention, activity and impulsivity in dogs based on owner report.

## Competing interests

The authors declare that they have no competing interests.

## Authors' contributions

LL conceptualized this study, designed the survey, conducted primary data analyses, and drafted the manuscript. JBS contributed to survey design, aided in data analyses and interpretation and assisted with manuscript preparation. AMI supervised data analyses and assisted with manuscript preparation. AMO contributed to survey design, assisted with data analyses and manuscript preparation. All authors have given approval of the final manuscript.

## Supplementary Material

Additional file 1 **Survey questions**. Questions derived from the ADHD Rating Scale [47] and Vas et al. [40], modified for American dog owners. Click here for file

Additional file 2 **Breed frequencies**. Frequencies of owner-reported breeds included in the study. Click here for file

Additional file 3 **Subscales**. Questions derived from the ADHD Rating Scale [47] and Vas et al. [40], modified for American dog owners, and divided according to subscale identified in this study. Click here for file
