# Supplementary material for: Owner reports of attention, activity, and impulsivity in dogs: a replication study
Source: Behav Brain Funct. 2010 Jan 4;6:1. doi: 10.1186/1744-9081-6-1 (PMC2823640; doi:10.1186/1744-9081-6-1)
Supplement: Additional file 1 — Survey questions. Questions derived from the ADHD Rating Scale [47] and Vas et al. [40], modified for American dog owners. [file 1744-9081-6-1-S1.PDF]

Additional file 1. Questions from the ADHD Rating Scale (DuPaul, 1998) and Vas et al. (2007), modified for American dog owners.

1. Your dog has a difficult time learning, because other things can easily attract its attention (ie, easily distracted)
2. It's easy to attract your dog's attention, but your dog loses interest easily.
3. It's difficult for your dog to concentrate on a talk or on play.
4. It's difficult for your dog to maintain a stay.
5. Your dog barks endlessly, seemingly at nothing important.
6. Your dog fidgets all the time or is in constant motion.
7. Even if your dog knows someone is speaking to him/her, your dog doesn't seem to pay any attention.
8. Your dog likes active play and running around.
9. Your dog often has difficulties performing tasks, even if your dog has practiced them often.
10. Your dog is likely to react hastily or anticipate.
11. Your dog's attention can be easily distracted.
12. Your dog cannot wait as it has no self-control.
