# Supplementary material for: Owner reports of attention, activity, and impulsivity in dogs: a replication study
Source: Behav Brain Funct. 2010 Jan 4;6:1. doi: 10.1186/1744-9081-6-1 (PMC2823640; doi:10.1186/1744-9081-6-1)
Supplement: Additional file 2 — Breed frequencies. Frequencies of owner-reported breeds included in the study. [file 1744-9081-6-1-S2.PDF]

Additional file 2. Frequencies of owner-reported breeds included in the study.

Breed	Frequency
Not Stated	10
Airedale	20
Akita	2
Alaskan Malamute	2
American Bulldog	1
American Cocker Spaniel	11
American Dingo - Carolina Dog	1
American Eskimo	1
American Pit Bull Terrier	2
American Staffordshire Terrier	1
Australian Cattle Dog	9
Australian Kelpie	3
Australian Shepherd	21
Australian Shepherd Mini	3
Azawakh	1
Basenji	1
Basset Hound	6
Beagle	3
Bearded Collie	1
Beauceron	2
Bedlington Terrier	2
Belgian Malinois	11
Belgian Sheepdog	2
Belgian Tervuren	7

Bichon Frise	6
Black and Tan Coonhound	1
Bloodhound	6
Blue Heeler	1
Border Collie	48
Border Terrier	7
Borzoi	1
Boston terrier	3
Bouvier de Flanders	1
Boxer	5
Boykin Spaniel	1
Briard	1
British Staffordshire Bull Terrier	1
Brittany Spaniel	3
Bullmastiff	2
Cairn Terrier	2
Canaan Dog	1
Catahoula Leopard Dog	1
Cavalier King Charles Spaniel	2
Chesapeake Bay Retriever	1
Chihuahua	3
Chinese Crested	1
Chinese Shar Pei	10
Chow Chow	1

Clumber Spaniel	1
Collie Rough	57
Collie Smooth	11
Coton de Tulear	7
Curly Coated Retriever	1
Dachsund	11
Dingo	1
Doberman Pinscher	8
Dogo Argentino	1
Dogue de Bordeaux	1
Dutch Shepherd	4
English Bulldog	1
English Cocker Spaniel	2
English Mastiff	1
English Pointer	1
English Shepherd	1
English Springer Spaniel	12
Field Spaniel	1
Finnish Spitz	1
Flatcoated Retriever	3
Fox Terrier	1
German Shepherd	52
German Shorthaired Pointer	4
Golden Retriever	95
Great Dane	4

Great Pyrenees	2
Greyhound	4
Havana Silk	17
Hovawart	1
Ibizan Hound	1
Irish Setter	4
Irish Water Spaniel	14
Irish Wolfhound	1
Italian Greyhound	10
Jack Russell Terrier	5
Keeshond	1
Kerry Blue Terrier	1
Komondor	2
Kuvasz	2
Labrador Retriever	47
Manchester Terrier	8
Mastiff	2
Miniature Pinscher	5
Miniature Schnauzer	1
Mixed	205
Newfoundland	3
Norwegian Elkhound	3
Norwich Terrier	1
Old English Sheepdog	1
Papillon	1

Parson Russell Terrier	1
Pekinese	1
Pit Bull	4
Pomeranian	3
Poodle	3
Poodle Miniature	8
Poodle Standard	44
Poodle Toy	2
Portuguese Water Dog	1
Pug	4
Pumi	2
Pyrenees Shepherd	1
Rat Terrier	4
Redbone Coon Hound	1
Rhodesian Ridgeback	5
Rottweiler	19
Saint Bernard	1
Saluki	1
Samoyed	2
Schnauzer Giant	1
Shetland Sheepdog	20
Shih Tzu	2
Siberian Husky	5
Soft Coated Wheaten Terrier	3
Springer Spaniel	1
Staffordshire Bull Terrier	1

Standard Schnauzer	6
Sussex Spaniel	1
Tibetan Terrier	6
Treeing Walker Coonhound	2
Vizsla	1
Welsh Corgi Cardigan	2
Welsh Corgi Pembroke	7
Welsh Springer Spaniel	1
West Highland Terrier	1
Whippet	3
Wire Fox Terrier	1
Yorkshire Terrier	3
Total	1030
