# Supplementary material for: Owner reports of attention, activity, and impulsivity in dogs: a replication study
Source: Behav Brain Funct. 2010 Jan 4;6:1. doi: 10.1186/1744-9081-6-1 (PMC2823640; doi:10.1186/1744-9081-6-1)
Supplement: Additional file 3 — Subscales. Questions derived from the ADHD Rating Scale [47] and Vas et al. [40], modified for American dog owners, and divided according to subscale identified in this study. [file 1744-9081-6-1-S3.PDF]

Additional file 3. Questions from the ADHD Rating Scale (DuPaul, 1998) and Vas et al. (2007), modified for American dog owners, and divided according to subscale identified in this study.

Inattention Subscale

1. Your dog has a difficult time learning, because other things can easily attract its attention (ie, easily distracted)
2. It's easy to attract your dog's attention, but your dog loses interest easily.
3. It's difficult for your dog to concentrate on a talk or on play.
7. Even if your dog knows someone is speaking to him/her, your dog doesn't seem to pay any attention.
9. Your dog often has difficulties performing tasks, even if your dog has practiced them often.
11. Your dog's attention can be easily distracted.

Hyperactivity-Impulsivity 1 Subscale

4. It's difficult for your dog to maintain a stay.
5. Your dog barks endlessly, seemingly at nothing important.
6. Your dog fidgets all the time or is in constant motion.
12. Your dog cannot wait as it has no self-control.

Hyperactivity-Impulsivity 2 Subscale

6. Your dog fidgets all the time or is in constant motion.
8. Your dog likes active play and running around.
10. Your dog is likely to react hastily or anticipate.
